# Singular Case of Triplets

**Published:** 1857-06

**Authors:** 


*Singular Case of Triplets.*—Dr. A. S. McGregor, of Gasconade Ferry, Mo., in a letter to the editor of this journal, says, on the 10th of August, 1856, Mrs. G——, of this county, was delivered of a still born child, twenty-one days after this she gave birth to a second, and in the same length of time thereafter of a third The two last lived about six hours each. The mother is doing well, and is again pregnant.—*Ibid.*

